# Preventive Effect of *Ecklonia cava* Extract on DSS-Induced Colitis by Elevating Intestinal Barrier Function and Improving Pathogenic Inflammation

**DOI:** 10.3390/molecules28248099

**Published:** 2023-12-15

**Authors:** Young-Mi Kim, Hye-Youn Kim, Ji-Tae Jang, Suntaek Hong

**Affiliations:** 1 Lee Gil Ya Cancer and Diabetes Institute, Department of Biochemistry, Gachon University College of Medicine, Incheon 21999, Republic of Korea; zieziebe@hanmail.net (Y.-M.K.); 01199296307@hanmail.net (H.-Y.K.); 2 Aqua Green Technology Co., Ltd., Smart Building, Jeju Science Park, Jeju 63309, Republic of Korea; jtjang@aquagt.co.kr

**Keywords:** *Ecklonia cava* extract, DSS-induced colitis, intestinal barrier, pathogenic inflammation

## Abstract

Inflammatory bowel disease (IBD), including ulcerative colitis and Crohn’s disease, is a complex gastrointestinal disorder with a multifactorial etiology, including environmental triggers, autoimmune mechanisms, and genetic predisposition. Despite advancements in therapeutic strategies for IBD, its associated mortality rate continues to rise, which is often attributed to unforeseen side effects of conventional treatments. In this context, we explored the potential of *Ecklonia cava* extract (ECE), derived from an edible marine alga known for its anti-inflammatory and antioxidant properties, in mitigating IBD. This study investigated the effectiveness of ECE as a preventive agent in a murine model of dextran sulfate sodium (DSS)-induced colitis. Our findings revealed that pretreatment with ECE significantly ameliorated colitis severity, as evidenced by increased colon length, reduced spleen weight, and histological improvements demonstrated by immunohistochemical analysis. Furthermore, ECE significantly attenuated the upregulation of inflammatory cytokines and mediators and the infiltration of immune cells known to be prominent features of colitis in mice. Notably, ECE alleviated dysbiosis of intestinal microflora and aided in the recovery of damaged intestinal mucosa. Mechanistically, ECE exhibited protective effects against pathogenic colitis by inhibiting the NLRP3/NF-κB pathways known to be pivotal regulators in the inflammatory signaling cascade. These compelling results suggest that ECE holds promise as a potential candidate for IBD prevention. It might be developed into a functional food for promoting gastrointestinal health. This research sheds light on the preventive potential of natural compounds like ECE in the management of IBD, offering a safer and more effective approach to combating this challenging disease.

## 1. Introduction

Inflammatory bowel disease (IBD), a composite of ulcerative colitis (UC) and Crohn’s disease (CD), comprises a cluster of protracted inflammatory conditions affecting the gastrointestinal tract. These disorders, characterized by chronic and unbridled inflammation alongside epithelial deterioration of the intestinal mucosa, ensue from a complex interplay of genetic predisposition and a milieu of environmental risk factors [1,2,3]. Although the precise mechanisms of IBD’s pathogenesis remain to be discovered, an emerging consensus implicates that a disordered immune modulation within the gastrointestinal microenvironment and a concomitant breakdown in the homeostasis of the epithelial barrier might play important roles in IBD [4]. Untreated IBD carries a marked elevation in the risk of colorectal cancer, underscoring the imperativeness of efficacious IBD management [5].

During the development of IBD, many pathological phenomena are accompanied by defective changes in intestinal environments. Due to excess inflammatory stimuli, immunological dysregulation is initiated with damage to the epithelial barrier, infiltration of immune cells, and dysbiosis of intestinal flora [6,7]. Activated intestinal cells are known to express high levels of inflammatory cytokines to accelerate pathological processes. The intestinal epithelial barrier serves to protect the host by blocking the entry of pathogenic microorganisms and foreign antigens into the body. It is composed of enterocytes that are tightly connected through intercellular junctions [8]. Intestinal barrier dysfunction in IBD refers to uncontrolled inflammation due to increased intestinal permeability, decreased tight junction (TJ) barrier function, and impaired immune regulation [9,10]. Mucin 2 (MUC2) secreted by goblet cells can also prevent colonization by pathogenic microorganisms and the transfer of enterotoxins from bacteria to the internal environment [11]. With a defective mucosal barrier, compositions of the intestinal microbiome can change into those of dysbiotic strains. These pathogenic bacteria can induce more destructive immune responses by interacting with Toll-like receptor to activate inflammatory signaling through nucleotide-binding oligomerization domain-like receptor protein 3 (NLRP3) and NF-κB-mediated signal transduction [12,13].

Traditional IBD therapeutics, from antibiotics to biologics and immunosuppressants, are frequently accompanied by a series of significant side effects [14]. The recent direction of IBD etiological research has ushered in the potential for innovative therapeutic modalities, including naturally occurring compounds. These compounds proffer the prospect of redressing perturbations in the gut microbiome while simultaneously expediting the restorative process of the mucosal layer [15,16]. Among such natural compounds, complex marine polysaccharides have been extensively studied as pharmaceuticals [17,18]. Based on previous reports, algae extracts and polysaccharides are excellent substances for treating and preventing intestinal inflammation such as IBD due to the anti-inflammatory functions they provide as fermentation substrates for beneficial intestinal microbiomes [19,20]. Among this category, *Ecklonia cava* (*E. cava*), an edible brown alga distributed along the coasts of Korea, China, and Japan, is composed of various physiologically active substances such as fucoidan, sulfated polysaccharide, and phlorotannin [21,22]. Compared to other brown algae, *E. cava* is rich in a unique polyphenol with polymerized phloroglucinol units called phlorotannin, among various components. Phlorotannins, including dieckol (DK), eckol, and phlorofucofuroeckol A (PFFA) in *E. cava*, are known to exhibit many biological potentials against viral infection, diabetic complications, hypertension, and obesity-associated phenomena [23,24,25,26]. These phlorotannins in *E. cava* suggest that they can be used as a natural good source with potential applications in various diseases [27,28]. 

Studies on *E. cava*’s anti-inflammatory properties have been conducted in several fields. In a periodontal disease-related study, *E. cava* prevented alveolar bone loss by reducing inflammatory cell infiltration and IL-1β production in gingival tissue, and it also attenuated endothelial cell dysfunction by regulating the inflammation of perivascular adipose tissue in cardiovascular disease [29,30]. It has also been reported that it can be used as a preventative and therapeutic compound for diabetes-related diseases by reducing inflammation-associated receptors such as TLR4 and RAGE [31]. Despite extensive investigations into these anti-inflammatory activities, the specific role of *E. cava* in IBD remains a largely unexamined area. 

Thus, we tried to assess the preventive efficacy of *E. cava* extract (ECE) using a colitis animal model employing various colitis disease activity indices. Ultimately, we aim to illuminate the potential of ECE as a safe and efficient agent for promoting intestinal health, thus paving the way for its development as a functional food without adverse long-term effects.

## 2. Results

### 2.1. Ecklonia cava Extract Protects against DSS-Induced Colitis

**Figure 1:**
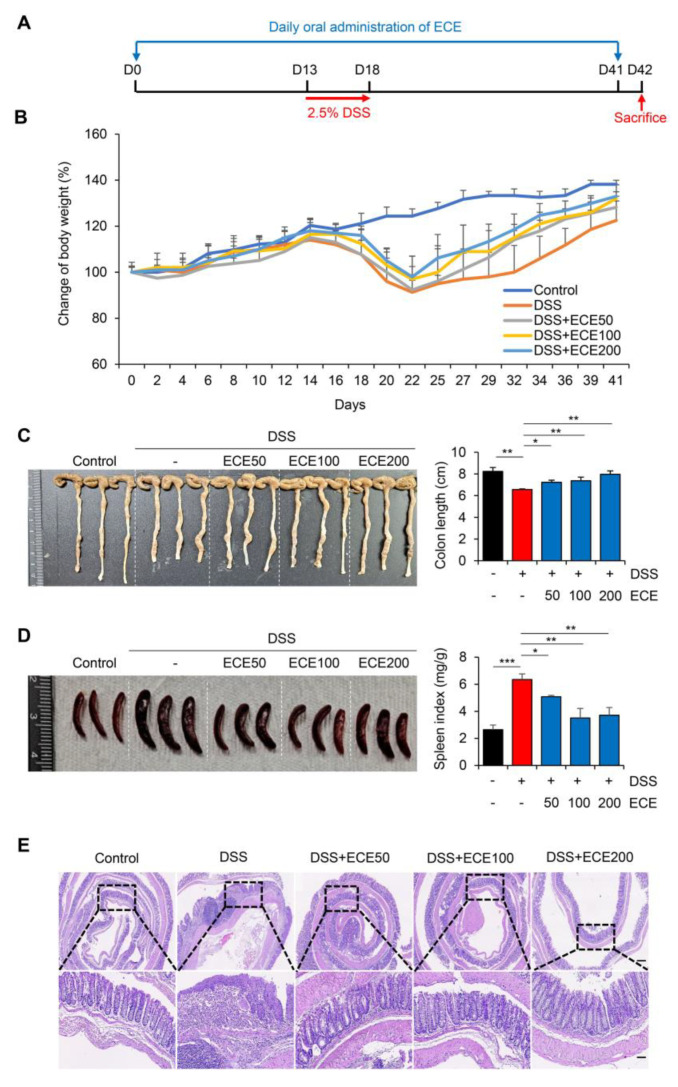
*Ecklonia cava* extract protects against dextran sodium sulfate (DSS)-induced colitis: (**A**) Experimental design for DSS-induced colitis and *Ecklonia cava* extract (ECE) pretreatment by oral administration. Different doses (50, 100, 200 mg/kg body weight) of ECE were orally administrated to mice every day until the end of experiment. After 14 days, mice were fed with drinking water containing 2.5% DSS for 5 days. Mice were sacrificed at 42 days after treatment to analyze disease activity index. (**B**) Body weight changes in control or DSS-treated mice and ECE-pretreated colitis mice. (**C**) Gross morphology of colon (left) and quantification of colon length (right). Colon length was measured, except for the cecum. (**D**) Representative image of spleen (left) and quantitative analysis of spleen weight-to-body weight ratio. (**E**) Representative image of H&E-stained colon tissue. Scale bar = 300 μm (top, 4×), 60 μm (bottom, 20×). All *p*-values were calculated using unpaired two-tailed Student’s *t*-tests. Results are presented as mean ± SD from at least triplicate samples. *, *p* < 0.05; **, *p* < 0.01; ***, *p* < 0.001.

To determine whether ECE could prevent colonic damage and inflammation, we prepared a dextran sulfate sodium (DSS)-induced colitis model (Figure 1A). ECE was orally administered daily from 14 days before DSS treatment until mice were sacrificed. Mice were fed 2.5% DSS in drinking water for 5 days. After that, the mice were fed normal drinking water. Weight loss was confirmed in the DSS group compared to the normal group. There was a little restoration of body weight between the DSS group and the ECE-treated groups in a dose-dependent manner (Figure 1B). Colon length, an indicator of severity of colitis, was found to be longer in the ECE-treated group than in the DSS group (Figure 1C). Moreover, the spleen index (spleen weight/body weight) was markedly increased in the DSS group compared to in the normal group. However, it was significantly restored in the ECE-treated groups (Figure 1D). Histopathological examination of the colon revealed that colonic crypt damage and mucosal infiltration of immune cells in the DSS group were improved in the ECE-treated groups (Figure 1E).

To determine a preventive effect of ECE against colitis with therapeutic potential, an animal model was prepared by orally administering ECE after DSS treatment (Figure S1A). In contrast with the preventive model, body weight change was not significant between the DSS and ECE groups during therapeutic treatment (Figure S1B). Colon length and spleen index were not dramatically changed by treatment with ECE (Figure S1C,D). Consistent with phenotypical changes, hematoxylin–eosin (H&E) staining showed no difference in submucosal damage or destruction of colonic structure between the DSS and ECE groups (Figure S1E). These data suggest that ECE is more effective against DSS-induced colitis through preventive use rather than as a therapeutic treatment.

### 2.2. Ecklonia cava Extract Reduces Inflammatory Response in DSS-Induced Colitis

**Figure 2:**
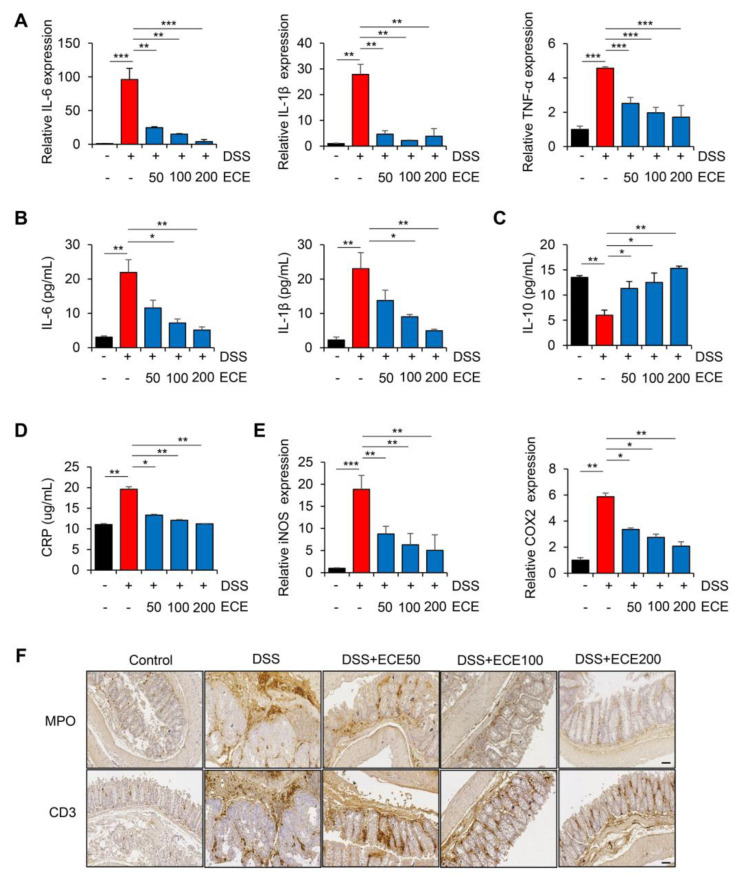
*Ecklonia cava* extract reduces inflammatory response in DSS-induced colitis: (**A**) IL-6, IL-1β, and TNF-α mRNA expression levels in colon tissues were analyzed using real-time PCR. GAPDH was used as a control for normalization. (**B**) Measurements of IL-6 and IL-1β protein levels in serum using ELISA kit. (**C**) Measurement of IL-10 protein levels in serum using ELISA kit. (**D**) ELISA quantification of CRP concentration in serum. (**E**) COX2 and iNOS mRNA expression levels in colon tissues were analyzed using real-time PCR. GAPDH was used as a normalization control. (**F**) Representative images of immunostaining of MPO and CD3 in colon tissues. IHC scores of MPO and CD3 were quantified using Image J software (version 1.37). Scale bar = 60 μm. All *p*-values were calculated using unpaired two-tailed Student’s *t*-tests. Results are presented as mean ± SD from at least triplicate samples. *, *p* < 0.05; **, *p* < 0.01; ***, *p* < 0.001.

DSS-induced colitis is known to be closely associated with the production of pro-inflammatory cytokines [32]. To determine whether the DSS-induced expression of pro-inflammatory cytokines was affected by ECE, we first measured levels of pro-inflammatory cytokines in colon tissues and sera samples. As shown in Figure 2A, mRNA levels of pro-inflammatory cytokines such as IL-6, IL-1β, and TNF-α in colon tissues were increased in DSS-treated mice but significantly suppressed by ECE in a dose-dependent manner. It was also confirmed that protein levels of IL-6 and IL-1β in sera samples were decreased in the ECE-treated groups (Figure 2B). Notably, pretreatment with ECE enhanced levels of the anti-inflammatory cytokine IL-10 in the sera of DSS-treated mice (Figure 2C).

C-reactive protein (CRP), another inflammatory cytokine, is synthesized in the liver in response to tissue damage, microbial infection, and autoimmune diseases [33]. IL-6 and IL-1β are known to strongly induce CRP expression. They were reported to be increased in a DSS-induced colitis mouse model [34,35]. As expected, the serum CRP concentration increased by DSS was significantly decreased in the ECE-treated groups to normal levels (Figure 2D). Moreover, mRNA levels of iNOS and COX2, two inflammatory enzymes increased by DSS, were decreased in colon tissues of the ECE-treated groups (Figure 2E). 

It was found that there is a positive relationship between colon inflammation and increased immune cell infiltration in a DSS-induced colitis mouse model [36,37]. Therefore, we performed immunohistochemical (IHC) staining of colon tissue to determine whether ECE could affect immune cell infiltration. Severe infiltration of T cells (CD3) and neutrophils (MPO) appeared in DSS-treated mice. However, they were significantly reduced by ECE in a dose-dependent manner (Figure 2F and Figure S2). These data suggest that ECE has an anti-inflammatory effect on DSS-induced colitis by suppressing the production of pro-inflammatory mediators and the infiltration of immune cells by changing the intestinal environment.

### 2.3. Ecklonia cava Extract Ameliorates Gut Microbiome Imbalance with DSS-Induced Colitis

The gut microbiome is involved in immune homeostasis and gut maintenance. Thus, it has been of great interest in IBD research and biologic therapy in recent years [38]. Several studies have shown that compositions of the gut microbiome are different between people with IBD and those without IBD, particularly regarding the abundance and diversity of certain bacteria [39,40]. The destruction of gut microbiome homeostasis in patients with colitis is characterized by dysbiosis, which can decrease beneficial microorganisms such as *Firmicutes* bacteria and increase harmful microorganisms such as *Bacteroidetes* bacteria [41,42].

**Figure 3:**
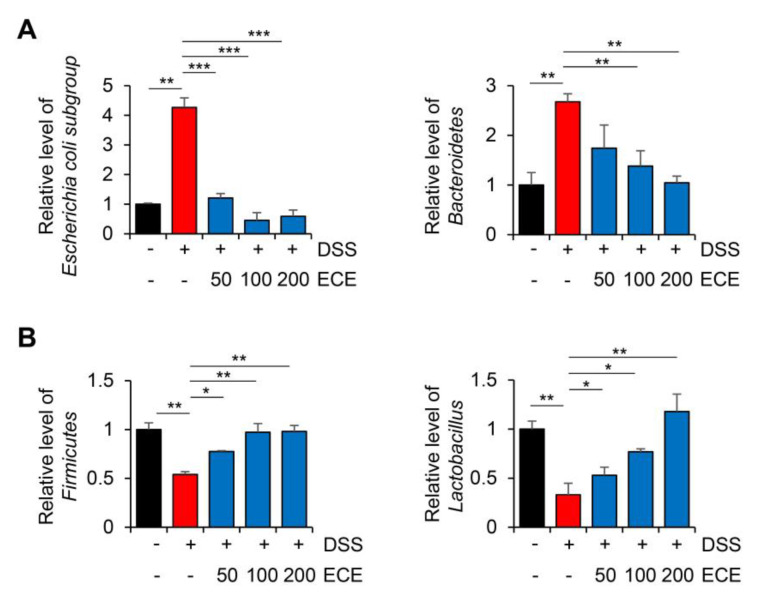
*Ecklonia cava* extract ameliorates gut microbiome imbalance in mice with DSS-induced colitis. DNA was extracted from the cecum of each group and used as a template. Real-time PCR was then performed. The relative abundance of bacterial groups (**A**); beneficial bacterial group (**B**); harmful bacterial group) is expressed as a percentage of eubacteria. All *p*-values were calculated using unpaired two-tailed Student’s *t*-tests. Results are presented as mean ± SD from at least triplicate samples. *, *p* < 0.05; **, *p* < 0.01; ***, *p* < 0.001.

To determine whether ECE could modulate the distribution of the gut microbiome in DSS-induced colitis, the relative level of intestinal microbiota was determined using cecum 16S rRNA-specific PCR. As shown in Figure 3, in mice with DSS-induced colitis, an imbalance of the gut microbiome was observed with an increase in harmful microbiomes and a decrease in beneficial ones. Compared with the DSS-treated group, the ECE-treated groups showed a decreased abundance of the *Escherichia coli* subgroup and *Bacteroidetes*. In addition, the abundance of *Firmicutes* and *Lactobacillus*, which had been reduced by DSS, was significantly increased in the ECE-treated groups. These results reveal that ECE could ameliorate intestinal dysbiosis in DSS-induced colitis by modulating the balance between beneficial bacteria and harmful ones.

### 2.4. Ecklonia cava Extract Restores Stability of Intestinal Barrier

**Figure 4:**
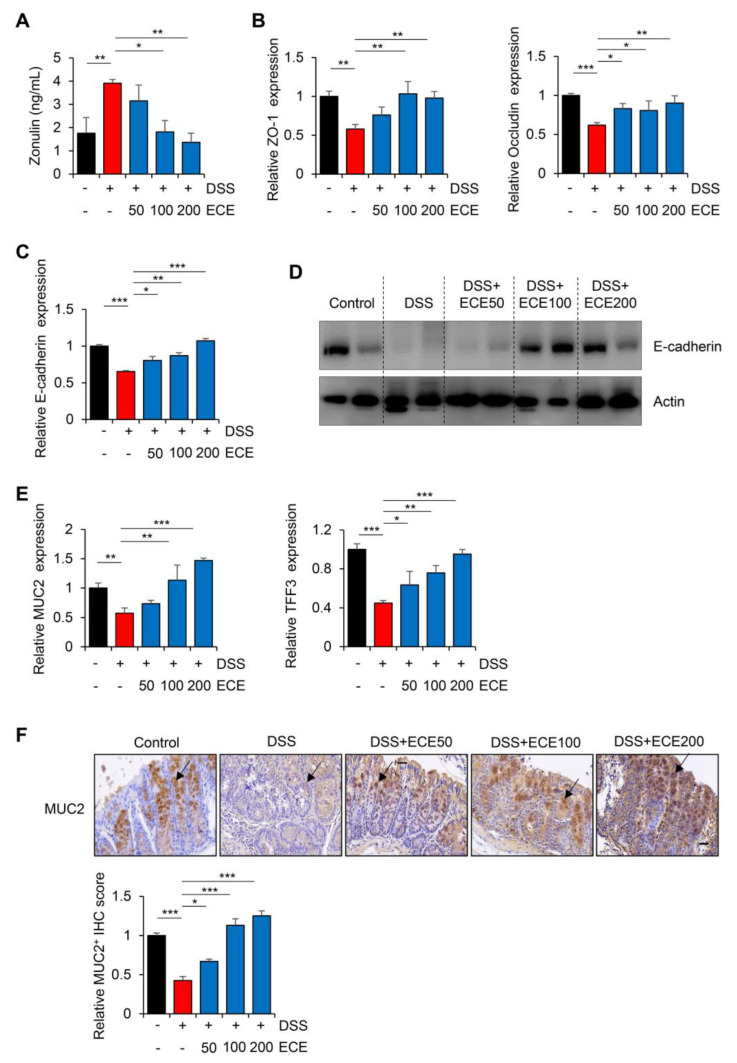
*Ecklonia cava* extract restores stability of intestinal barrier: (**A**) Determination of Zonulin protein level in mouse serum using ELISA kit. (**B**) mRNA quantification of tight junction proteins (ZO-1 and occludin) in colon tissues was performed using real-time PCR. GAPDH was used as a normalization control. (**C**,**D**) Comparison of E-cadherin expression in colon tissue was performed using real-time PCR and Western blot. GAPDH and actin were used as normalization controls, respectively. (**E**) mRNA quantification of MUC2 and TFF3 in colon tissues was performed using real-time PCR. GAPDH was used as a normalization control. (**F**) Representative immunohistochemical staining images (top) and IHC quantification with Image J software (version 1.37) (bottom) of MUC2 in colon tissues. Black arrow indicates the stained MUC2 protein. Scale bar = 30 μm. All *p*-values were calculated using unpaired two-tailed Student’s *t*-tests. Results are presented as mean ± SD from at least triplicate samples. *, *p* < 0.05; **, *p* < 0.01; ***, *p* < 0.001.

The intestinal barrier functions to maintain mucosal homeostasis by filling in the gap between the intestinal immune system and intestinal microbes [43]. Zonulin, a marker of barrier integrity, can reversibly increase intestinal permeability by modulating tight junctions between cells [44,45]. To investigate the effect of ECE on the intestinal barrier integrity of mice with DSS-induced colitis, the protein concentration of Zonulin was measured in serum with an ELISA kit. As shown in Figure 4A, serum levels of Zonulin, which were increased in the DSS-treated group, were significantly reduced by ECE in dose-dependent manner.

DSS can also elevate intestinal permeability by disrupting epithelial cell tight junctions and adhesive junctions, thereby reducing mucus level [46,47]. Therefore, expression levels of ZO-1 and occludin, which are TJ proteins, and E-cadherin, which is an adhesion molecule, in colon tissues were examined. As expected, decreased mRNA expression levels of TJ proteins and E-cadherin by DSS were restored in the ECE-treated groups (Figure 4B,C). Consistent with restoration of mRNA level, the reduced protein expression of E-cadherin in the DSS-treated group also recovered in the ECE-treated groups (Figure 4D).

Among the mucins constituting the mucus layer that acts as a barrier against harmful substances in the intestine, the recovery of the expression level of MUC2, which forms a gel only in the colon, is an indicator of improvement in colitis [48]. TFF3 expressed in goblet cells of the colon is known to protect the mucous membrane from damage and stabilize the mucosal layer [49]. Thus, the expression levels of MUC2 and TFF3 in colon tissues were determined by real-time PCR and IHC staining, respectively. As shown in Figure 4E, the expression levels of MUC2 and TFF3, which are related to MUC2 secretion, were markedly restored by ECE treatment. Moreover, IHC staining showed that the protein expression of MUC2 was decreased in the DSS-treated group, whereas its level in the ECE-treated group recovered to a level similar to that in the control group (Figure 4F). Taken together, these results suggest that the preventive effect of ECE on the intestinal epithelium might be derived from improvements in barrier function through a restoration of mucosal protection-related genes damaged by DSS-induced colitis. 

### 2.5. Ecklonia cava Extract Suppresses the Pathological Inflammatory Signaling

**Figure 5:**
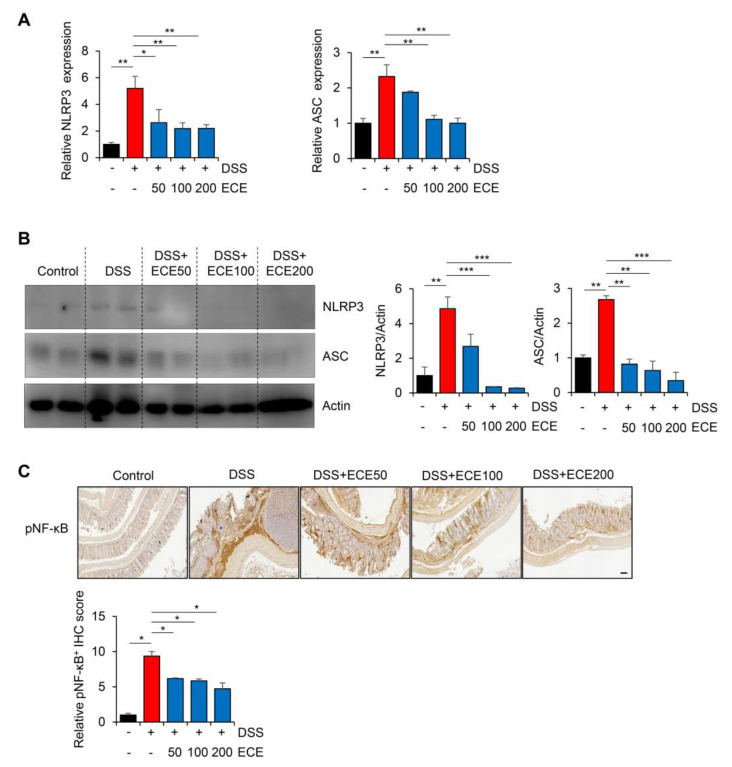
*Ecklonia cava* extract suppresses NLRP3 inflammasome and NF-κB pathway. (**A**) mRNA quantification of NLRP3 and ASC in colon tissue was checked using real time PCR. GAPDH was used as a normalization control. (**B**) Protein expression levels of NLRP3 and ASC in colon tissues were evaluated by Western blot (top). Intensity of Western blot band was quantified using ImageJ (bottom). Actin was used as normalization control. (**C**) Representative images of pNF-κB immunostaining (top) in colon tissues are presented and relative staining levels (bottom) are quantified using ImageJ software (version 1.37). Scale bar = 100 μm. All *p*-values were calculated using unpaired two-tailed Student’s *t*-tests. Results are presented as mean ± SD from at least triplicate samples. *, *p* < 0.05; **, *p* < 0.01; ***, *p* < 0.001.

The NLRP3 inflammasome is an intracellular complex that can induce inflammation in IBD. Its expression is increased by DSS [50,51]. In addition, it has been reported that the NLRP3 inflammasome is inhibited by ECE in non-alcoholic fatty liver disease and muscle atrophy [52,53]. To determine whether ECE could affect the NLRP3 inflammasome pathway in DSS-induced colitis, we measured NLRP3 activation in colon tissues using qRT-PCR and Western blot, respectively. The results showed that the expression levels of NLRP3 and ASC mRNAs and proteins were increased in the DSS-treated group, but attenuated in the ECE-treated groups (Figure 5A,B).

Next, we confirmed activation of NF-κB, which increased NLRP3 expression, through IHC analysis. As shown in Figure 5C, phosphorylation of NF-κB, which was increased in the DSS-treated group, was dramatically decreased by ECE in a dose-dependent manner. These data suggest that ECE can prevent colitis-induced changes in intestinal permeability, microbiota distribution, and inflammatory markers by modulating the activity of upstream NLRP3 and NF-κB mediators.

## 3. Discussion

**Figure 6:**
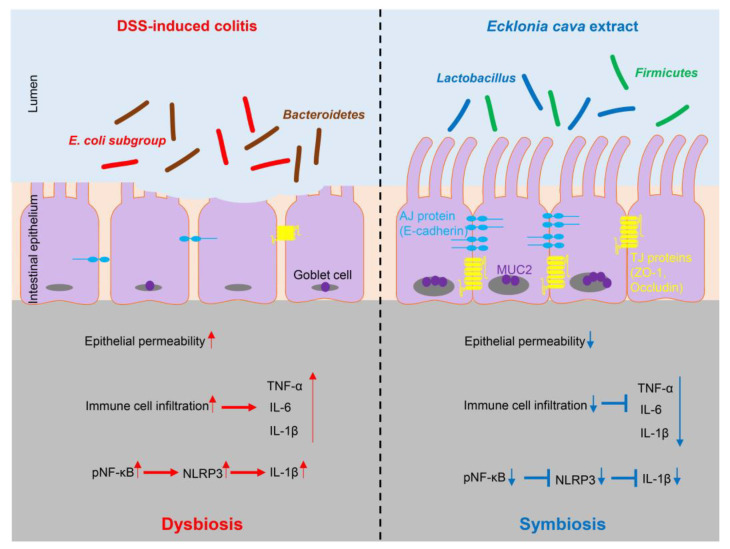
Protective effect of *Ecklonia cava* extract on DSS-induced colitis. In colitis, an imbalance of microorganisms can lead to invasion of harmful bacteria and loss of proteins involved in barrier integrity (such as ZO-1 and E-cadherin) and increase epithelial permeability. In addition, upregulation of inflammatory cytokines can lead to infiltration of immune cells and an increase in IL-1β secretion by NLRP3 inflammasome activation. In contrast, preventive administration of ECE can promote the recovery of beneficial bacteria and improve intestinal bacteria imbalance. ECE can prevent loss of TJ and AJ proteins and increase expression of MUC2, consequently reducing epithelial permeability. Furthermore, ECE can suppress the expression of inflammatory cytokines and inhibit NLRP3 inflammasome activation. Taken together, these results demonstrate that ECE can maintain intestinal homeostasis by changing the intestinal microenvironment into a highly immune-enhanced one. Red arrows indicate the increase or activation of gene expression or pathway and blue arrows indicate the decrease or suppression of gene expression or pathway.

In our study, pretreatment with ECE alleviated the severity of colitis by improving colon shortening, reducing spleen weight gain, and mitigating histological damage in DSS-induced colitis (Figure 1). The balance between pro-inflammatory cytokines, including IL-6, IL-1β, and TNF-α, usually plays a crucial pathological role in colitis by mediating inflammatory responses [32]. We observed a significant reduction in the increase in pro-inflammatory cytokines induced by DSS in ECE-treated mice. Conversely, serum levels of IL-10, an anti-inflammatory cytokine acting as a negative regulator in colitis, were increased by ECE. Additionally, T cell and neutrophil infiltration were notably reduced in ECE-treated mice (Figure 2). The gut microbiome significantly influences gut health, and mucosal-associated bacteria directly impact the integrity of the intestinal epithelial barrier layer by increasing the mucus layer’s thickness and promoting intestinal barrier repair. Several studies report that an imbalance of commensal bacteria is closely associated with the development of various diseases such as IBD [38,39,40]. Our results revealed an increased abundance of colitis-associated *Escherichia coli* subgroup and *Bacteroidetes* species, alongside a relative decrease in *Firmicutes* and *Lactobacillus* (Figure 3). Tight junctions are pivotal in maintaining the integrity of the intestinal epithelial barrier, crucial for intestinal homeostasis. A disruption of tight junctions and epithelial permeability are linked with IBD progression [44]. Pretreatment with ECE reduced the concentration of Zonulin, a factor increasing barrier permeability, and restored the expression of tight junction proteins (ZO-1, occludin) (Figure 4A,B). The mucus layer covering the mucosal surface of the intestinal lumen, a protective gel-like substance composed of mucin secreted by goblet cells, is associated with colitis development when disrupted [48]. Our experiment revealed a significant reduction in MUC2 expression in the DSS-treated group, which was restored in the ECE-treated groups (Figure 4F). The NLRP3 inflammasome significantly contributes to the onset and progression of IBD. Overexpression of the NLRP3 inflammasome exacerbates colitis and plays a pivotal role in intestinal inflammation in DSS-induced colitis [50,51]. Our data exhibited NLRP3 activation after DSS-induced colitis; however, ECE pretreatment reduced NLRP3 expression and further suppressed NF-κB activation, a prerequisite for NLRP3 activation (Figure 5). In summary, our study elucidated the preventive efficacy of ECE in the context of IBD using a DSS-induced colitis mouse model. ECE pretreatment resulted in a reduced inflammatory response attributed to the downregulation of NLRP3/NF-κB signaling. Furthermore, ECE demonstrated the capacity to enhance both barrier function and microbiome homeostasis (Figure 6).

IBD is a chronic digestive disease accompanied by recurrent inflammation due to complex causes such as genetic, microbial, and environmental factors. Its prevalence is rapidly increasing worldwide [54]. Currently, conventional drugs used to treat IBD encompass anti-inflammatory drugs, immunosuppressants, and glucocorticoids. Although anti-TNF-α drugs have shown high efficiency in IBD management, they are associated with the risk of infectious complications and allergic reactions. In addition, their efficacy may wane over time [55]. Oral 5-aminosalicylic acid-based drugs such as olsalazine, sulfasalazine, balsalazide, and mesalazine can also cause nausea and headaches, while corticosteroids are linked to adverse effects such as hypertension, exacerbation of gastric ulcers, and osteoporosis [56]. In parallel, advanced therapeutic modalities, including small-molecule drugs with economical profiles, convenient administration, and biotherapeutics with heightened effectiveness driven by specific mechanisms, are under development. However, these interventions are not devoid of undesirable side effects [57]. Considering these serious side effects, finding new sources to improve clinical symptoms of IBD is still essential. Notably, IBD patients experience compromised quality of life. They are burdened by inflammatory phenotypes, prompting comprehensive exploration into adjunctive therapies such as probiotics, dietary interventions, polyphenols, and microbial metabolites [58]. A promising avenue involves the investigation of natural compounds capable of expediting restoration of the intestinal mucosal layer and normalizing the gut microbiome. Achieving these involves impeding leukocyte infiltration into inflamed intestinal mucosa and curtailing the secretion of inflammatory cytokines, thus presenting innovative approaches to address IBD pathogenesis [59].

The gut microbiome is a key factor in gut health. Mucosal-associated bacteria are directly related to the integrity of the intestinal epithelial barrier layer by increasing the thickness of the mucus layer and promoting intestinal barrier repair. Perturbations in commensal microbial balance have been closely linked to the onset and progression of diverse diseases including IBD [60]. Noteworthy among these is the genus *Lactobacillus*, comprising beneficial probiotic microorganisms recognized for generating antibiotic compounds that can hinder colonization by pathogenic bacteria while suppressing the production of pro-inflammatory cytokines such as IL-1β, IL-6, and TNF-α. This action orchestrates favorable shifts in the compositions of intestinal flora [61]. Recent discoveries have unveiled the potential of select probiotics as potent anti-inflammatory mediators in IBD, acting by restoring gut microbiota composition to alleviate and prevent intestinal disorders [62].

Presently, the bulk of research concerning intestinal health has predominantly investigated extracts sourced from terrestrial origins. Examples include dietary fibers and extracts derived from compounds such as curcumin and *Rhodiola crenulata*, which have demonstrated potential in preventing colitis by mitigating inflammatory cytokine secretion and sustaining intestinal barrier integrity [63,64]. In contrast, the oceanic realm, an immense repository of natural components, offers an underexplored frontier. Seaweeds characterized by their polyphenolic, proteinaceous, and polysaccharide constituents have gained scientific attention. Seaweed polyphenols highlighted for their antioxidant and antiviral attributes are subject to ongoing investigation for therapeutic applications. Furthermore, seaweed polysaccharides exhibit diverse physiological functions, including anti-inflammatory, antioxidant, antiviral, and immunomodulatory effects, fueling active research into their potential therapeutic utility [20,65].

Notably, various alga extracts have demonstrated significant efficacy in a colitis mouse model. Extracts from *Saccharina japonica*, a brown macroalga, suppressed inflammatory signaling caused by DSS, altering intestinal microbial diversity and regulating the intestinal microenvironment, thereby alleviating inflammatory bowel disease symptoms [66]. Additionally, an extract from the green alga *Ulva pertusa* effectively reduced tissue damage and suppressed an inflammatory response induced by DNBS [67]. These findings underscore the potential of diverse marine alga extracts to manifest anti-inflammatory and anticolitis effects. Moreover, the extensive research substantiating these effects necessitates continued exploration of compounds derived from alga extracts [16].

Dieckol, a major phlorotannin derivative isolated from *E. cava* and rich in polysaccharides and polyphenols, has undergone extensive study regarding its antiallergic and antioxidant properties [68,69]. In in vitro anti-inflammatory studies, dieckol upregulates hemeoxygenase-1 (HO-1), mediating anti-inflammatory effects in macrophages, and inhibits PI3 K and AKT phosphorylation in colon cancer cells, thereby impeding cancer cell proliferation and migration [70,71]. Furthermore, in a DSS-induced ulcerative colitis model, dieckol’s effectiveness in suppressing inflammation was verified by activating the Nrf2 and HO-1 signaling pathways [72]. Overall, dieckol exhibits anti-inflammatory properties and demonstrates efficacy when administered alone, although its effects may be amplified when combined with other anti-inflammatory drugs.

As previously mentioned, multiple studies have highlighted ECE’s anti-inflammatory properties [29,30,31]. Our findings suggest its potential as an additive in functional foods designed to fortify the intestinal immune environment against inflammatory bowel disease. Additionally, considering the effectiveness of ECE in combination treatments [73], it could be paired with other drugs to enhance its efficacy in inflammatory bowel disease. Moreover, a precise understanding of the active ingredient through synthesizing analogs resembling ECE’s active components could lead to the development of more potent drugs for IBD prevention [22].

## 4. Materials and Methods

### 4.1. Reagents

ECE was supplied from Aqua Green Technology Co., Ltd. (Jeju, Republic of Korea) and dissolved in phosphate-buffered saline for experiments. To make ECE, *E. cava* was washed and dried at room temperature for 48 h. After 50% (*v*/*w*) ethanol was added, it was incubated at 85 °C for 12 h. The extract was then filtered, concentrated, sterilized by heating to over 85 °C for 1 h, and then dried for use as described previously [23,74,75]. DSS (36–50 kDa) was purchased from MP Biomedicals (Santa Ana, CA, USA).

### 4.2. Characterization of Ecklonia cava Extract Using High-Performance Liquid Chromatography Analysis and Toxicity of Ecklonia cava Extract

High-performance liquid chromatography (HPLC) analysis was performed using a Waters HPLC system (Waters, Framingham, MA, USA) equipped with a 2998 photodiode array (PDA) detector, 2707 autosampler, and 515 HPLC pump. The C18 column (4.6 × 100 mm, 4 μm, Agilent, Santa Clara, CA, USA) was used for separation. For the analysis of ECE, solvent A (methanol) was used as the mobile phase and solvent B (water) was used as the stationary phase. The ECE was eluted using a gradient of solvent A and solvent B at a flow rate of 0.3 mL/min. The gradient method was as follows: 0 min 63:37 *v*/*v*; 0–5 min 63:37–63:37 *v*/*v*; 5–10 min 50:50 *v*/*v*; 10–20 min 35:65 *v*/*v*; 20–25 min 63:37 *v*/*v*; and 25–35 min 63:37 *v*/*v*. The absorption spectra were analyzed using a PDA detector at 230 nm range. Pure DK was used as standard marker for quantification (Figure S3).

A 14-day acute oral toxicity test was conducted using the prepared ECE, during which no mortalities were observed among the animals administered the extract. General observations, including body weight, revealed no abnormal symptoms. Furthermore, post-mortem examination did not indicate any abnormalities in any of the administered groups.

### 4.3. DSS-Induced Colitis Mouse Model

To induce colitis, 6-week-old C57BL/6 male mice (Orient Bio, Seongnam, Republic of Korea) were fed drinking water containing 2.5% DSS for 5 days. They were then fed normal drinking tap water. To prepare a preventive model, mice in the ECE-treated colitis group (50, 100, 200 mg/kg body weight) were preadministered with ECE through an oral gastric gavage 2 weeks before the start of DSS administration, and this was administered daily until sacrifice. ECE doses were determined based on concentrations used in previous studies. The selected dose fell within a range containing the minimum level of dieckol, the active ingredient of ECE [23,35,75,76]. In therapeutic experiments, ECE was administered for 3 weeks after DSS treatment. Body weight was measured every 2–3 days. On days 42 or 28, mice were euthanized using CO_2_ gas inhalation for 90 s followed by placement in a box containing CO_2_ gas for 4 min. The colon and spleen were separated, photographed, and weighed. Colon tissue was fixed in formalin for paraffin sectioning and immediately stored in liquid nitrogen for RNA and protein extraction. All mouse experiments adhered to guidelines approved by the Institutional Animal Care and Use Committees of Gachon University (AAALAC-accredited facility, approval number LCDI-2022-0046).

### 4.4. Western Blot

Total proteins from colon tissues were lysed with NP buffer (50 mM Tris-HCl (pH 7.5), 150 mM NaCl, 5 mM EDTA, 1% NP-40, and a protease/phosphatase inhibitor cocktail) for 30 min on ice and centrifugated at 13,000 rpm for 10 min at 4 °C. Protein concentration was measured using a BCA kit (Thermo Fisher Scientific, Rockford, IL, USA). BSA was used as a standard. Protein samples were boiled for 5 min at 100 °C in sample buffer (60 mM Tris-HCl (pH 6.8), 14.4 mM 2-mercaptoethanol, 2% SDS, 0.05% bromophenol blue, 25% glycerol) and separated by SDS-PAGE. Proteins were transferred to methanol-activated PVDF membranes. Membranes were blocked with 5% skim milk in TBST for 1 h at room temperature. After washing the membrane with TBST, primary antibodies were added and incubated overnight at 4 °C. The following day, blots were incubated with HRP-conjugated goat anti-secondary antibodies for 1 h at room temperature followed by chemiluminescence detection (Atto, Amherst, NY, USA) [77]. Primary antibodies are shown in Table S1.

### 4.5. Total RNA Isolation and Quantitative Real-Time PCR

Total RNA was isolated using TRIzol reagent (Invitrogen, Carlsbad, CA, USA) and 1 μg mRNA was transcribed to cDNA with random hexamers using PrimeScript 1st strand cDNA synthesis kit (Takara, Japan). SYBR-green Premix Ex-Tag II (Takara, Kyoto, Japan) was used for quantification of cytokine transcripts with real-time quantitative PCR on a Prism 7900HT sequence detection system (Thermo Fisher Scientific). PCR results were analyzed using the comparative 2^−ΔΔCT^ method using GAPDH as a control [78]. Experiments were performed in triplicate and expressed as mean ± standard deviation (SD). Primer sequences used for qRT-PCR are shown in Table S2.

### 4.6. Bacterial DNA Extraction from Mice Ceca and Microbiota Analysis

After mice were sacrificed, contents of their ceca were immediately placed in liquid nitrogen and frozen at −80 °C until use in experiments. Bacterial DNA was extracted using a DNA stool extraction kit (Qiagen, Valencia, CA, USA) according to the manufacturer’s instructions. Then, 10 ng bacterial DNA was used as a template for PCR [79]. The 16S rRNA of each group was analyzed with bacterial-strain-specific RT-PCR primers. The relative abundance of a bacterial group in the cecal samples was expressed as a ratio of eubacteria. Primer sequences used for RT-PCR are listed in Table S3.

### 4.7. ELISA for Serum Markers

Concentrations of IL-6, IL-1β, IL-10, CRP (R&D systems, Minneapolis, MN, USA), and Zonulin (MyBioSource, San Diego, CA, USA) in mouse serum samples were evaluated using ELISA kits according to the manufacturer’s protocol [77]. Briefly, blood samples were allowed to clot for 2 h at room temperature to collect serum. After centrifugation at 2000× *g* for 20 min, serum aliquots were stored at −0 °C before use. ELISA kits used in this study are listed in Table S4.

### 4.8. Hematoxylin–Eosin Staining and Immunohistochemistry

Paraffin-embedded tissues were sectioned at a thickness of 3 μm and stained with hematoxylin–eosin according to published procedures [80]. Briefly, tissue sections were deparaffinized with xylene, put in antigen retrieval buffer (Tris-EDTA buffer, pH 9.0), and boiled for 5 min. Endogenous peroxidase was blocked using 0.3% hydrogen peroxide. Slides were incubated overnight at 4 °C with primary antibodies diluted in 1% BSA followed by incubation with a secondary antibody for 1 h. For visualization, DAB substrate (Dako, Glostrup, Denmark) was used and counterstained with hematoxylin (Vector Laboratories, Burlingame, CA, USA). The image was captured using a confocal microscope at the Core-facility for Cell to In-vivo imaging of Gachon University and quantified using Image J software (version 1.37, NIH, Bethesda, MD, USA) [81]. Primary antibodies used for staining are shown in Table S1.

### 4.9. Statistics

Comparisons between groups were determined using Student’s *t*-test (two-tailed). The error bar represents the SD of the mean. Data are presented as mean ± SD. For all statistical tests, statistical significance was considered when the *p*-value was less than 0.05.

## 5. Conclusions

Our study revealed that ECE pretreatment likely suppressed the expression of inflammatory factors and increased intestinal barrier integrity by inhibiting the NLRP3/NF-κB pathway, resulting in a restoration of barrier dysfunction and reduced pathological inflammation. Our findings suggest that ECE can be developed as an intestinal health functional ingredient for preventive purposes or health products in combination with probiotics and other supplements.

## Data Availability

Data are contained within the article and Supplementary Materials.

## Supplementary Materials

The following supporting information can be downloaded at https://www.mdpi.com/article/10.3390/molecules28248099/s1: Figure S1: Therapeutic treatment of ECE shows less effective activity in DSS-induced colitis model; Figure S2: ECE efficiently suppresses infiltration of immune cells; Figure S3: Characterization of Ecklonia cava extract using high-performance liquid chromatography analysis. Table S1: List of primary antibody used in this study; Table S2: List of primer sequences used for real-time PCR; Table S3: List of primer sequences used for microbiota analysis; Table S4: List of ELISA kits used in this study.
