# Supplementary material for: Preventive Effect of Ecklonia cava Extract on DSS-Induced Colitis by Elevating Intestinal Barrier Function and Improving Pathogenic Inflammation
Source: Molecules. 2023 Dec 15;28(24):8099. doi: 10.3390/molecules28248099 (PMC10745772; doi:10.3390/molecules28248099)
Supplement: Supplementary file 1 [file molecules-28-08099-s001.zip › molecules-2757487-supplementary.pdf]

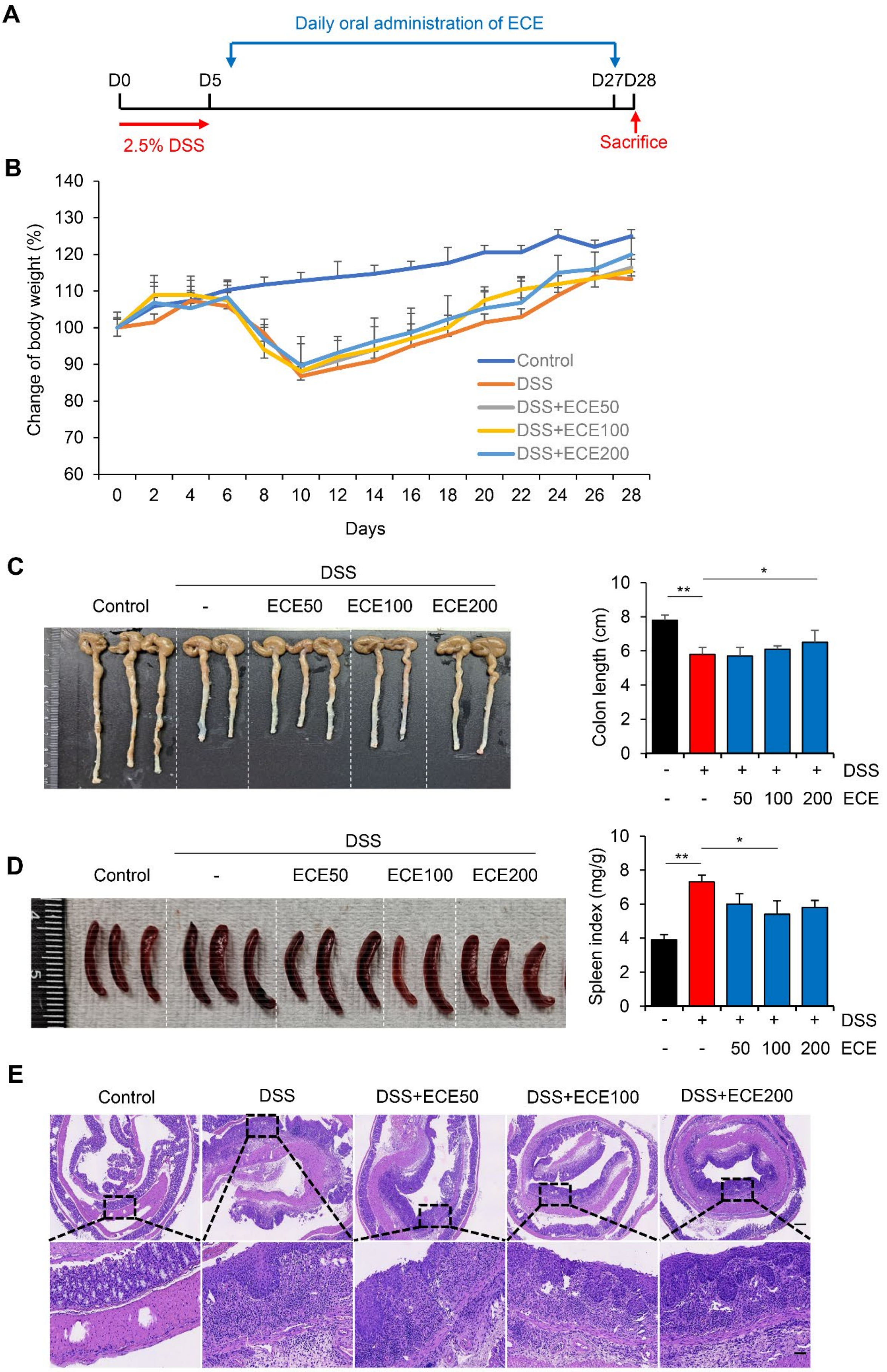
A
Daily oral administration of ECE
D0
D5
D27D28
2.5% DSS
Sacrifice
B
Change of body weight (%)
Days
Control
DSS
DSS+ECE50
DSS+ECE100
DSS+ECE200
C
DSS
Control
-
ECE50
ECE100
ECE200
Colon length (cm)
DSS
ECE
D
DSS
Control
-
ECE50
ECE100
ECE200
Spleen index (mg/g)
DSS
ECE
E
Control
DSS
DSS+ECE50
DSS+ECE100
DSS+ECE200

Figure S1. **Therapeutic treatment of ECE shows less effective activity in DSS-induced colitis model.** (A) Experimental design for DSS-induced colitis model and therapeutic treatment with ECE. Briefly, colitis was induced by feeding 2.5% DSS in drinking water for 5 days, followed by feeding with normal drinking water. In ECE-treated groups, ECE was orally administered after treatment with DSS. It was administered until the end of experiment. (B) Body weight changes during DSS-induced colitis experiment in control and ECE-treated mice. (C) Gross morphology of colon (left) and quantification of colon length (right). Colon length was measured except for the cecum. (D) Representative image of spleen (left) and quantitative analysis of the ratio of spleen weight to body weight. (E) Representative images of H&E stained colon tissues in DSS-induced colitis model with therapeutic treatment of ECE. Scale bar = 300 μm (top), 60 μm (bottom). All *P*-values were calculated using unpaired two-tailed Student's *t*-tests. Results are presented as mean ± SD from at least triplicate samples. *, $p < 0.05$; **, $p < 0.01$.

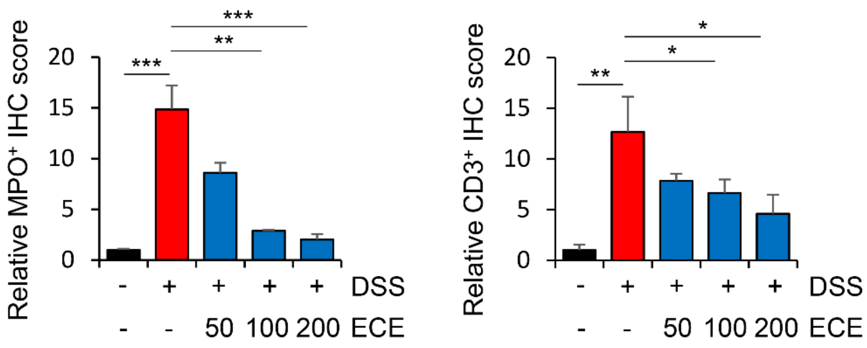

Figure S2. ECE efficiently suppresses infiltration of immune cells. The relative ratio of infiltrated immune cells was compared after capturing stained images with ImageJ software (related to Fig. 2F). The intensity was normalized against untreated control images. All *P*-values were calculated using unpaired two-tailed Student's *t*-tests. Results are presented as mean ± SD from at least triplicate samples. *, $p < 0.05$; **, $p < 0.01$; ***, $p < 0.001$.

A

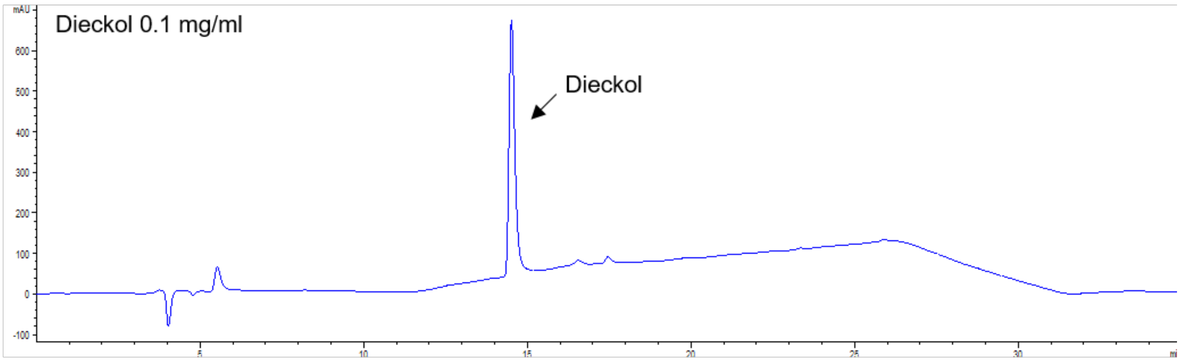

RT	Area
14.796	11319.6

B

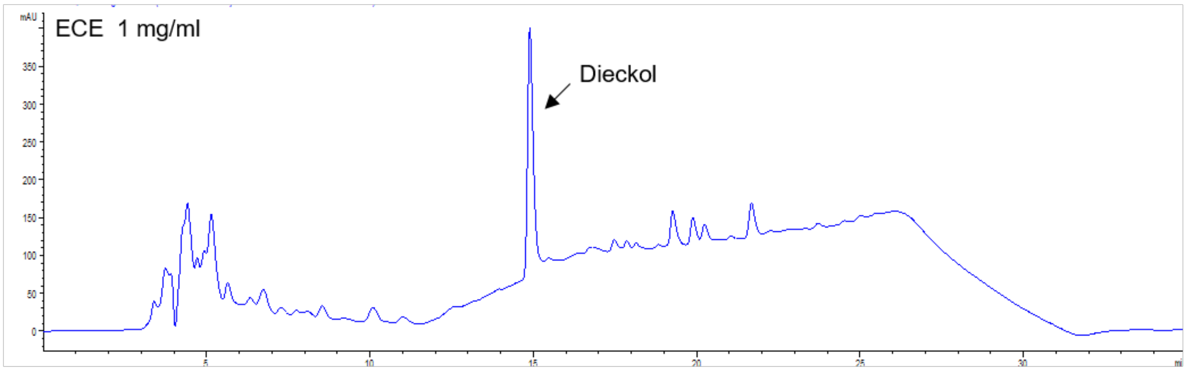

RT	Area	Dieckol (%)
14.896	3419.6	3.05±0.04

Figure S3. Characterization of *Ecklonia cava* extract using high-performance liquid chromatography analysis. HPLC analysis was performed with a methanol/water gradient at a flow rate of 0.3 ml/min using C18 column in Waters system. (A) The peak indicates dieckol (DK) as standard sample. The table shows the DK concentration compared to peak area. (B) The peak area for DK was calculated and confirmed over 3% DK as major bioactive ingredient within *Ecklonia cava* extract.

Table S1. List of primary antibody used in this study

Antibody	Company	Catalog number	Dilution rate
MPO	Abcam	ab208670	1:100 (IHC)
CD3	ThermoFisher	11-0031-82	1:100 (IHC)
pNF-κB	Cell signaling	3033	1:100 (IHC)
E-cadherin	BD biosciences	610182	1:1,000 (Western blot)
Actin	Sigma Aldrich	A5441	1:3,000 (Western blot)
MUC2	GeneTex	GTX100664	1:100 (IHC)
NLRP3	Abcam	214185	1:1,000 (Western blot)
ASC	Santa cruz	271054	1:1,000 (Western blot)

Table S2. List of primer sequences used for real time PCR

Primer	Sequence
IL-6	F: 5'-GAGGATACCACTCCCAACAGACC-3'
	R: 5'-AAGTGCATCATCGTTGTTCATACA-3'
IL-1β	F: 5-AGGCAGGCAGTATCACTCATTGT-3'
	R: 5'-GGAAGGTCCACGGGAAAGAC-3'
TNF-α	F: 5'-CATGACATCTGGCTTCACTC-3'
	R: 5'-TGGGAGTAGACAAGGTACAACCC-3'
iNOS	F: 5'-CGAAACGCTTCACTTCCAA-3'
	R: 5'-TGAGCCTATATTGCTGTG-3'
COX2	F: 5'-TGAAGACGTCCTCCACTCATG-3'
	R: 5'-CCTGGGATGGCATCAGTTT-3'
ZO-1	F: 5'-GCGCGGAGTTTCGGGT-3'
	R: 5'-AAACCCAGGAGCCCTGTGAA-3'
Occludin	F: 5'-TGAAAGTCCACCTCCTTACAGA-3'
	R: 5'-CCGGATAAAAAGAGTACGCTGG-3'
E-cadherin	F: 5'-GGCTGGCTGAAAGTGACACA-3'
	R: 5'-ACGGCATGAGAATAGAGGATGTACT-3'
MUC2	F: 5'-CTCTGCTGTCTCCGTCAT-3'
	R: 5'-CACTGGTCTTCTCCTCCTT-3'
TFF3	F: 5'-TCCAAGCCAATGTATGGTGCCG-3'
	R: 5'-CAGGGCACATTTGGGATACTGG-3'
NLRP3	F: 5'-GACCATCGGCCGGACTAAA-3'
	R: 5'-CGTCCTCGGGCTCAAACA-3'
ASC	F: 5'-GCACAGGCAAGCACTCATTG-3'
	R: 5'-CACACTGCCATGCAAAGCAT-3'
GAPDH	F: 5'-ATCTTCTTTTGCGTCGCCAG-3'
	R: 5'-CGTTGACTCCGACCTTCACC-3'

Table S3. List of primer sequences used for microbiota analysis

Primer	Sequence
Eubacteria	F: 5'-AAACTCAAAKGAATTGACGG-3'
	R: 5'-CTCACRRCACGAGCTGAC-3'
Escherichia coli subgroup	F: 5'-CATGCCGCGTGTATGAAGAA-3'
	R: 5'-CGGGTAACGTCAATGAGCAAA-3'
Bacteroidetes	F: 5'-GTTTAATTCGATGATACGCGAG-3'
	R: 5'-TTAASCCGACACCTCACGG-3'
Firmicutes	F: 5'-GGAGYATGTGGTTTAATTCGAAGCA-3'
	R: 5'-AGCTGACGACAACCATGCAC-3'
Lactobacillus	F: 5'-CGATGAGTGCTAGGTGTTGGA-3'
	R: 5'-CAAGATGTCAAGACCTGGTAAG-3'

Table S4. List of ELISA kit used in this study

ELISA kit	Company	Catalog number
Mouse IL-6	R&D systems	M6000B
Mouse IL-1β	R&D systems	MLB00C
Mouse IL-10	R&D systems	M1000B
Mouse CRP	R&D systems	MCRP00
Mouse Zonulin	MyBioSource	MBS2603528
